# Development of Seed-Oil Based Dried Sausages, Considering Physicochemical and Nutritional Quality and the Role of Food Neophobia

**DOI:** 10.3390/nu14153106

**Published:** 2022-07-28

**Authors:** Laura Tarjuelo, José Emilio Pardo, Manuel Álvarez-Ortí, Arturo Pardo-Giménez, Cristina Millán, Adrián Rabadán

**Affiliations:** 1 E.T.S.I. Agrónomos y de Montes, University of Castilla-La Mancha, Campus Universitario s/n, 02071 Albacete, Spain; laura.tarjuelo@alu.uclm.es (L.T.); jose.pgonzalez@uclm.es (J.E.P.); manuel.alvarez@uclm.es (M.Á.-O.); cristina.millan@uclm.es (C.M.); 2 Centro de Investigación, Experimentación y Servicios del Champiñón (CIES), C/Peñicas s/n, Apdo. 63, Quintanar del Rey, 16220 Cuenca, Spain; apardo.cies@dipucuenca.es

**Keywords:** poppy seeds, melon seeds, chia seeds, pumpkin seeds, food by-product

## Abstract

A growing number of consumers now consider the consumption of processed meat products to be an essentially unhealthy habit. Hence, the reformulation of meat products is crucial. In this regard, the aim of this study is to reformulate “fuet”, a traditional Spanish dried sausage, by replacing the pork fat with emulsified seed oils (50–50%, 25–75% and 0–100%). Four seed oils were evaluated, including commercial seeds (poppy and chia) and other seeds considered subproducts (melon and pumpkin). Physical parameters, nutritional quality and consumer evaluation of the reformulated dried sausages were analyzed. Additionally, we considered the effects of food neophobia on consumer evaluation. The resulting fuets had a higher concentration of linoleic and linolenic acids, which varied according to the oil used. In the sensory analysis, non-neophobic consumers showed higher preference for the reformulated fuets, while all consumers gave their highest ratings to the fuets produced with pumpkin seed oil.

## 1. Introduction

Meat products are considered one of the main sources of dietary fat [1], which is considered a crucial factor in the development of severe chronic diseases [2]. Multiple studies have concluded that dietary fat increases the risk of obesity and the development of certain types of cancer [2,3]. Moreover, due to their high percentages of saturated fats, the consumption of meat products is also associated with high blood cholesterol and coronary heart disease [4,5]. Considering all this information, health organizations across the world have promoted lowering total fat intake [6,7,8]. The reduction of intake of red and processed meat has been explicitly proposed as a means of preventing different chronic and severe diseases, mainly derived from heart problems [9].

Although in some developed countries, such as Spain, meat consumption is tending to fall [10,11], the reduction is not as significant when the consumption of specific meat products is considered [11]. In this regard, meat and meat products are still projected to be among the main components of consumers’ shopping trolleys. To meet current consumer demand for meat products and, at the same time, protect consumers’ health, manufacturers should develop novel food products that meet consumer expectations, but using a healthier approach [12]. This requires the reformulation of meat products, even of more traditional ones [13].

As reported above, most of the problems derived from meat consumption are directly related to fat content and fat quality [4,7]. If the content of fat can be reduced or the type of fat can be replaced, the nutritional quality of these meat products can be greatly increased [14]. Under the first approach, fat-reduced meat products should be developed. According to Regulation (EC) No 1924, 1924/2006 of the European Parliament and of the Council, a product may be considered a fat-reduced product when it has at least 30% less fat than a similar product. It may additionally be considered an energy-reduced food if the total energy value is also decreased by at least 30%. However, the reduction of fat in food presents certain technical constraints in food processing and can even be rejected by consumers [15]. The content of fat in meat is crucial to improve tenderness, juiciness, and flavor [16]. Palatability, which is crucial for ensuring consumer acceptance, also depends on how much chemical fat is present in the meat and its appearance in the product [17].

To avoid consumer rejection, most studies have explored the second possibility: the substitution of animal fat with other healthier fats. In this regard, the total or partial replacement of animal fats with plant oils [5,18], marine oils [19] or seed and nut oils [14,20] has attracted most of the interest. Most of the proposed fat-substitute ingredients also show high percentages of fat, but their fatty acid profile mainly comprises unsaturated fatty acids (mainly oleic linoleic and linolenic fatty acids) [14,19]. This results in products with higher nutritional quality that provide similar texture and juiciness to those of the original meat products, but reducing the negative effects on health of saturated fats [20,21].

Among the traditional, fermented meat products elaborated in Mediterranean countries, the dry-fermented sausage is one of the most important products [22]. The sausage traditionally called “fuet” is a small-caliber non-acid fermented sausage made with pork meat and fat as the main ingredients. In fuet sausage, fat can account for up to 50% of the product [23], although lower levels of fat are commonly used [24]. Although this fat is a source of vitamins and essential fatty acids [22], its high energy value and high percentage of saturated fatty acids could discourage the consumption of this product [4,7]. In this regard, and with the purpose of ensuring that consumption of traditional fuet is still possible under a healthier approach, substantial efforts have been made to reformulate this product [22,25,26,27].

When fat content is reduced in fermented sausages, the final product is darker and harder [24,25], with important sensory properties, such as flavor and texture, also being affected [27]. All this would lead to a reduction in consumer acceptability. Better results have been obtained when pork fat is substituted by plant oils [22,26,28,29]. However, most of these studies have replaced only small amounts of animal fat or have used small amounts of vegetable oils. For example, Mora-Gallego, Serra, Guàrdia, Miklos, Lametsch and Arnau [26] and Mora-Gallego, Guàrdia, Serra, Gou and Arnau [22] used 1.5–5% of sunflower oil in their new formulas, while Pelser, Linssen, Legger and Houben [28] elaborated Dutch-style fermented sausages, replacing 10–20% of pork backfat with flaxseed and canola oils. Although these reformulations show the path to follow, further effort is needed to substantially decrease the presence of animal fat in the final product with the least possible effect on consumer acceptance.

Although a notable reformulation of the product seems the main path to follow, it should be considered that not all consumers are open to accepting new or novel foods. Some are highly reluctant to accept or enjoy new foods or accept the addition of new ingredients or the use of novel food production techniques [12,30]. This phenomenon has been defined as food neophobia [31]. Attending to the existing literature food neophobia can be regarded both as a trait (related to personality) or as a state (more variable and dependent on the food environment of the individual) [32]. In this regard, the systematic review developed by Rabadán and Bernabéu [32] suggests a progressive reduction in the level of food neophobia across countries, as food neophobia seems to decrease with increasing education, income, and urbanization.

To measure consumer attitudes towards new foods, Pliner and Hobden [33] developed the Food Neophobia Scale as an eight-item, 9-point Likert scale. In their study, Pliner and Hobden [33] found that food neophobia correlated negatively with foreign food familiarity, finickiness, and sensation seeking, and positively with other fear and anxiety measures [31]. As a result, the FNS accurately predicts consumer responses to new foods such as insects or genetically modified plants [31,32]. In recent years, the study of food neophobia has been used to evaluate consumer attitudes to novel or non-traditional products [31,32]. However, only a small number of studies have used the FNS when studying the acceptance of reformulated products [18,34], although some have reported that the acceptance of these products could be at least partially related to the degree of neophobia [14].

The aim of this study is to determine how replacing animal fat with seed oils affects the physical, nutritional, and sensory characteristics of traditional fuet sausages. Moreover, the effect of food neophobia as a factor determining the sensory evaluation of reformulated products by potential consumers is also considered.

## 2. Materials and Methods

The melon and pumpkin seeds were supplied by Vicente Peris S.A. (Albuixet, Valencia), a company that obtains these seeds as a subproduct of their production of ready-to-eat fruit and vegetables. The chia and poppy seeds were acquired from a local store (El Corte Inglés S.A., Albacete, Spain).

The pumpkin, chia, poppy, and melon seed oils were extracted using a hydraulic press (MECAMAQ model DEVF 80, MECAMAQ, El Palau D’Anglesola, Spain), with which high-quality virgin oils can be obtained [35]. Before pressing, the seeds were ground, using a knife mill (RETSCH model GM 20, knife diameter 118 mm, Retsch GmbH, Haan, Germany). The size of the obtained particles after grinding was below 300 µm. After extraction and to remove impurities, the oils were centrifuged at 12,000 rpm for five minutes, using a CENTRONIC-BL centrifuge (J.P. Selecta, Abrera, Spain). Once extracted, the oils were refrigerated and stored at 4 °C in opaque glass bottles until the fuet sausages were produced.

The meat used to prepare the batches of fuet was high-quality pork (70% pork shoulder and 30% pork belly) purchased in a local store (Carnes Soria, Albacete, Spain). All the intermuscular and subcutaneous fat was separated from the meat. The lean meat and fat were then ground.

All the sausages were formulated with 76% lean meat and 24% fat, while the control sausage was formulated with 76% lean mean and 24% pork fat. Following the aim of the study, in all the other sausage formulations, the fatty part was totally or partially replaced with emulsified seed oils (poppy, chia, pumpkin and melon). Three different proportions were used: 50% pork fat–50% seed oil, 25% pork fat–75% seed oil, and 0% pork fat–100% seed oil. Thirteen different fuet formulations were developed including the control sample and three different formulations for each oil. For the emulsification the oils, 420 g of each oil was mixed with guar gum (28 g), inulin (42 g) and water (910 g). Inulin is a prebiotic food fiber derived from fructose extracted from roots and tubers. The guar gum was used as a thickener. The emulsification of oils was made for triplicate for each type of oil [36]. Three independent batches of sausages of each formulation (control; 50–50%; 25–75%, 0–100%) using each type of emulsified oil (poppy, chia, pumpkin, and melon) were prepared.

The lean and the fatty parts, in the different proportions, were hand-mixed in batches and then stuffed into casings using a manual sausage stuffer. Once prepared, the fuet sausages were stored for three weeks in maturing chambers at 14 °C and 78% humidity. With the aim of the sausages being developed in an environment as similar as possible to that of the industry, they were manufactured at the “El Conchel” meat factory located in El Ballestero (Albacete), following the procedure typically used by this industry.

### 2.1. Physical Analysis

The color of the samples was measured using a Minolta CR-200 colorimeter (Minolta Camera Co. Ltd. Osaka, Japan; Measuring aperture: 8 mm; Illuminant: CIE D65; Observer angle: CIE 2° Standard Observer). Color parameters were measured by reflection in five random zones of the surface in six different samples of each formulation and the average of each sample was used in the statistical analysis. As recommended by the Commission Internationale de l’Eclairage [37], the tristimulus values obtained were used to calculate the CIELAB chromatic coordinates: L* (lightness), a* (red–green component), b* (yellow–blue component).

The texture of the dry sausages was analyzed using a texture analyzer TA-XT Plus (Stable Micro Systems, Godalming, United Kingdom) equipped with a 50-mm-diameter probe at 3.3 mm/s^−1^. Three samples in each of the formulations were analyzed and the average of each sample was used in the statistical analysis. The samples analyzed were compressed to 60% of their original height at room temperature. The Texture Profile Analysis (TPA) test is one of the main analytical tests to establish the quality of dry fermented sausages [38,39]. The parameters analyzed were hardness (peak force of first compression cycle), springiness (distance of the detected height of the fuet on the second compression divided by the original compression distance), cohesiveness (ratio of positive areas of second cycle to area of first cycle) and chewiness (hardness × cohesiveness × springiness). Additionally, stickiness was measured (force to overcome the attractive forces between the dry sausage and the probe).

### 2.2. Nutritional and Chemical Analysis

First, FA methyl esters were obtained by cold transmethylation with methanolic potassium hydroxide, by adding 2 mL *n*-hexane to 0.02 g oil previously extracted from the flour samples by Soxhlet extraction with petroleum ether (3 h) (EC, 2002) [40]. Then, 200 μL methanolic potassium hydroxide solution (2 mol L^−1^) was added and vigorously mixed. Once the supernatant was transferred to a glass vial and analyzed by gas chromatography in a Shimadzu GC-2010 Plus gas chromatograph equipped with a split–splitless injector, a flame ionization detector and an autosampler (AOC-20i, Shimadzu, Tokyo, Japan). A CPSil 88 fused-silica capillary column (Varian, Middelburg, The Netherlands; 50 m × 0.25 mm i.d., 0.20 μm film thickness) was used, and helium was used as gas carrier (120 kPa). The following temperature program was used: 5 min at 140 °C, followed by an increase of 5 °C min^−1^ from 140 to 220 °C and maintained at 220 °C for 15 min. The temperature of the injector and detector were 250 and 270 °C, respectively, and the split ratio was of 1:50 with an injection volume of 1 μL. Each fatty acid methyl ester (FAME) was identified by direct comparison with a standard mixture (FAME 37, Supelco, Bellefonte, PA, USA) [41]. The analyses were performed in triplicate for each formulation and the results expressed in relative percentage of each FA, based on the relative peak areas.

The nutritional components of the fuets were analyzed after grinding the samples. Three sausages in each of the formulations were analyzed. Following the method proposed by Lau [42], water content was measured as the loss of weight of the samples after being dried at 105 °C for 72 h. Protein content was calculated by multiplying the total nitrogen content previously calculated using the Kjeldahl method [43] by a conversion factor of 6.25. To determine ash content, flours were incinerated at 550 °C to constant weight. Crude fat content was estimated gravimetrically by the filter bag technique after petroleum ether extraction of the dried sample in an Ankom XT10 extraction system [44]. Additionally, to determine crude fiber content, we used the Weende method adapted to the filter bag technique. As described in ANKOM [45], the Weende method is useful to determine the organic residue remaining after digestion with sodium hydroxide and sulfuric acid solutions. Total carbohydrate content was calculated as the difference between the fuet weight and the content of all the previous compounds (water, ash, crude protein, and total fat) [46]. Finally, the carbohydrate content (nitrogen-free) was calculated by subtracting the fiber content from the total carbohydrate content [47].

### 2.3. Consumer Evaluation

Affective tests were used to assess consumer acceptance of the different fuet formulations proposed. The tests were conducted at the sensory analysis laboratory located at the Higher Technical School of Agricultural and Forestry Engineering in Albacete (Spain). A total of 101 consumers participated in the two affective tests undertaken. Two different sessions were held, and the same consumers participated in the evaluation of all samples. The consumers evaluated the physical aspect, texture, odor, and taste of each of the samples using a 9-point scale (−4: extremely dislike; 0: neither like nor dislike; +4: like extremely).

As well as the affective tests, the consumers completed a survey to establish their level of food neophobia, using the Food Neophobia Scale (FNS). This 10-item scale assesses consumer acceptance of new or innovative food products [32]. For the survey, we used the translation of the original FNS proposed by Fernández-Ruiz et al. [48]. The consumers were asked to indicate their level of agreement with each of the items, using a 7-point Likert-type scale ranging from “strongly disagree” (1) to “strongly agree” (7). The degree of neophobia was calculated as a value from 10 to 70 as the sum of all the items after reversing 1, 7, 9 and 10. The higher the score, the greater the food neophobia. The consumers were segmented into neophobics and non-neophobics using the median of the final value on the FNS for the consumers that participated in evaluating the reformulations with each of the oils.

Finally, the consumers reported the typical frequency of their fuet consumption according to four levels.

### 2.4. Statistical Analysis

The values presented are the mean scores with standard errors. To determine the color parameters, we used five measures to guarantee their quality, given the high level of variability in the results. The statistical differences between samples were estimated using variance analysis (ANOVA) at a 5% significance level, Duncan’s test (*p* < 0.05) and the T-test (*p* < 0.05). Pearson’s Chi^2^ test was applied to discrete variables. Variables considered for the Principal Component Analysis were factors related to color (L*, a*, b*, C*, h*), texture (hardness, stickiness, cohesiveness, springiness, chewiness), fatty acid composition (palmitic acid, stearic acid, oleic acid, linoleic acid, and linolenic acid) and nutrition evaluation values (content of protein, ashes, crude fat, total carbohydrates, and energy values). All statistical analyses were carried out using the SPSS program 23.0 for Windows (IBM, Armonk, NY, USA).

## 3. Results and Discussion

Table 1 shows the results obtained for the texture of the fuet samples prepared using emulsified seed oils. The control sample presented the highest hardness and cohesiveness values, with statistically significant differences in this respect being found between the control and the other samples. The previous literature has reported that hardness increases as fat content decreases [24,49]. Thus, substituting animal fat with a higher percentage of vegetable oil, including other ingredients and not only oil in its emulsification, might reduce hardness. In the study by Muguerza, Gimeno, Ansorena, Bloukas and Astiasarán [5], using pre-emulsified olive oil yielded products with lower values for hardness. Chewiness was also higher in the control sample, as in the studies by Rabadán, Álvarez-Ortí, Martínez, Pardo-Giménez, Zied and Pardo [14] and Mora-Gallego, Serra, Guàrdia, Miklos, Lametsch and Arnau [26], albeit with no significant differences from the emulsified pumpkin and melon seed oils.

Table 2 shows the results obtained for color. We analyzed L* (luminosity), a* (red-green component), b* (yellow-blue component), C* (chroma or purity) and h* (tone angle). The control sample yielded the lowest values for a*, b*, C* y h*, resulting in a color that was less yellow and less red, and a lower purity than the samples produced using emulsified seed oils. Papadima and Bloukas [50] reported that the percentage of fat in traditional Greek sausages determined their levels of L*, a* and b*. Nonetheless, the effects on product color of replacing animal fats with vegetable oils remains disputed. Our findings are consistent with those of Bloukas et al. [51], who concluded that replacing pork backfat with olive oil resulted in less yellow sausages. These authors, however, found higher L* values, while we found no statistically significant differences between the control sample and the reformulated sausages for this parameter. Meanwhile, Muguerza, Gimeno, Ansorena, Bloukas and Astiasarán [5], in a study on traditional Spanish fermented sausage (Chorizo de Pamplona), detected no clear effects of reformulation on the color of their samples.

**Figure 1:**
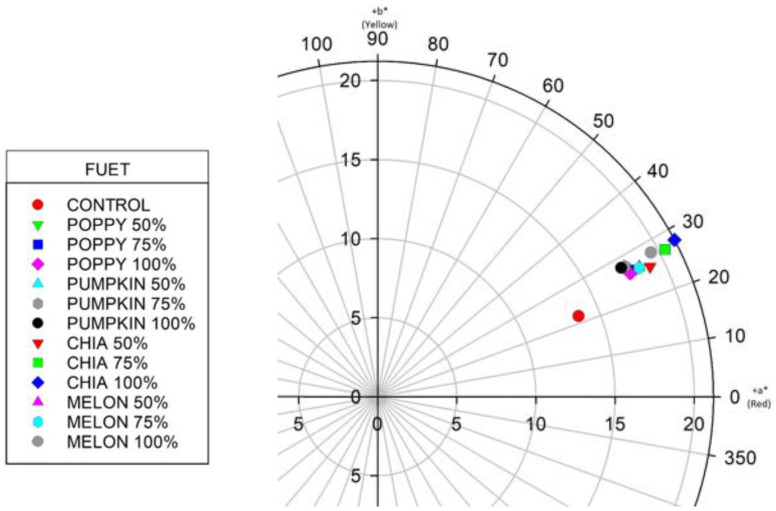
Graphical representation, in the CIELAB color space, of the results obtained for color of the fuet samples produced with different emulsified seed oils.

Figure 1 shows graphically, in the CIELAB color space, the color results obtained for the samples. The control sample is clearly distinct from the others, given its lower a* and b*values, while the values for the other samples differ little one from another. The sample produced with 100% chia seed oil is that with the most different values from those of the control, showing higher levels of red and yellow. Chia seed oil traditionally yields a yellow color (high b* values) [52]. The yellow differs in intensity according to the quantity of carotenoids in the oil, which, in turn, depends on the extraction process used [53]. Additionally, the samples including chia seed oil in their formulation also present significantly higher chroma values (C*).

Table 3 shows the results of the nutritional evaluation of the fuet samples. As reported in previous studies in which small percentages of animal fat were substituted with oils [54], no significant differences were found between samples for humidity and nitrogen, protein, and ash content. Significant differences were found, however, in crude fat and total carbohydrate content [55]. The control sample yielded the highest crude fat level (37.78%), being significantly different from the samples with a higher level of vegetable oil. The samples in which animal fat was 100% substituted with seed oil revealed a crude fat content of below 30%. It is worth noting that, in the study by [5], no differences in fat content were found between the control and the other samples, which could be accounted for by the lower percentage of substitution of animal fat used by these authors.

As reported by de Carvalho, Munekata, Pateiro, Campagnol, Domínguez, Trindade and Lorenzo [55], for each type of seed oil, we found that the crude fat content decreased as the percentage of oil replacing animal fat increased. This decline is attributed to the formula of oil emulsion being below 100%. Thus, higher percentages of substitutions result in oils with a lower fat content and a higher amount of total carbohydrates. However, although the control sample shows the highest energy value (555 kcal/100 g), the differences between this value and those of the other reformulated fuet sausages are not statistically significant. Consequently, replacing animal fat with vegetable oil does not serve to reduce the calorie content of the fuets, compared with the reformulations, in contrast to the use of cereal and fruit fiber [56], which is useful for producing low-fat dry fermented sausages.

Table 4 shows the results for the composition of main fatty acids of the dry sausage samples. The fatty acid composition obtained by substituting animal fat with seed oils differs from that reported in other studies replacing animal fat with olive oil, due to the different fatty acid profile of the oils used [5].

Other studies have shown that, even when small amounts of animal fat are replaced with other ingredients, such as oil of flour, the variations in unsaturated fatty acid content are substantial [20]. Monteiro, Souza, Costa, Faria and Vicente [54] detected significant differences in SFA, MUFA and PUFA content in Toscana sausage when substituting pork fat with canola oil at levels of 7.5%, 10% and 5%, respectively.

The control sample presented the highest saturated fatty acid values, specifically palmitic stearic acids. A higher presence of these fatty acids is considered a notable drawback, given that their consumption is associated with risks for human health [54,57], with this being one of the primary factors driving the reformulation of food products [36,58]. Our results show that reduction in the stearic acid content of the fuets was achieved with a 75% substitution of animal fat with poppy, pumpkin, and melon seed oils, while in the case of palmitic acid, it was necessary to replace 100% of the animal fat with poppy, melon and chia seed oils to generate significant reductions. In a study conducted by Muguerza, Gimeno, Ansorena, Bloukas and Astiasarán [5], no significant differences were found in the percentages of palmitic and stearic acids when substituting with emulsified olive oil up to a level of 30%, showing that higher levels of replacement are needed, such as those undertaken in the present work.

Regarding oleic acid (omega-9), the control sample also showed higher values than the reformulations, with this acid being progressively replaced by linoleic acid (omega-6) in the samples with the highest levels of substitution. This shift is due to the high proportion of linoleic acid present in the vegetable seed oils used [53,59], with the reformulations including larger percentages of substitution being those with the highest content of this fatty acid. The highest linoleic acid values were found in the fuet samples prepared with 100% substitutions of poppy, pumpkin, and melon seed oils, followed by the reformulations using the same three oils, but with a 75% level of substitution. The 50% substitutions were insufficient to generate significant changes in linoleic acid content. It has previously been reported that substituting 30% of animal fat with olive oil is insufficient to increase oleic and linoleic acid contents in other meat products [5].

As regards the level of linolenic acid (omega-3) in our samples, it should be noted that despite being residual in most of the fuet sausage formulations, it is an essential part of the fatty acid profile of those produced using emulsified chia seed oil, accounting for between 11.90% and 28.40% of the total, depending on the percentage of substitution. Using chia seed oil as a substitute for animal fat to enhance omega-3 content in meat products has been reported in previous studies, such as that by [60].

**Figure 2:**
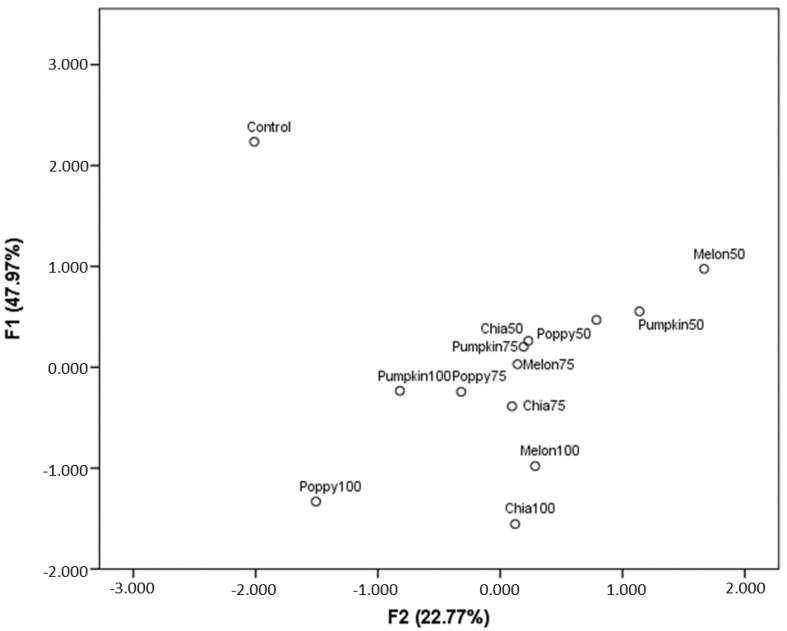
Principal component analysis (PCA) of different parameters in fuet samples. Color (L*, a*, b*, C*, h*) and texture (hardness, stickiness, cohesiveness, springiness, chewiness) parameters, fatty acid composition (palmitic acid, stearic acid, oleic acid, linoleic acid, and linolenic acid) and nutrition evaluation values (content of protein, ashes, crude fat, total carbohydrates, and energy value) are included. F1: L* (0.082), a* (−0.791), b* (−0.734), C* (−0.822), h* (−0.153), protein (−0.205), ashes (−0.694), crude fat (0.866), total carbohydrates (-0.888), energy value (0.866), palmitic (0.942), stearic (0.898), oleic (0.865), linoleic (−0.555), linolenic (−0.436), harness (0.781), stickiness (−0.408), cohesiveness (0.531), springiness (−0.167), chewiness (0.942). F2: L* (0.630), a* (0.075), b* (0.552), C* (0.212), h* (0.757), protein (−0.790), ashes (−0.504), crude fat (0.426), total carbohydrates (0.077), energy value (0.441), palmitic (0.201), stearic (0.372), oleic (0.251), linoleic (−0.356), linolenic (0.072), harness (−0.510), stickiness (−0.745), cohesiveness (−0.449), springiness (0.752), chewiness (−0.108).

Figure 2 shows the results of the PCA. The two linear combinations explain, overall, 70.74% of the variance that appears due to color and texture parameters, fatty acid composition and nutritional analysis. As expected, the control sample is very different to the reformulated fuets due to the differences in the color parameters (a*, b*, C*, h*), the fatty acid profile, and the values for hardness. Few differences are reported among samples in which 75% of the animal fat was replaced. However, differences increase severely when the replaced percentage increases up to 100%. When the animal fat is totally replaced with pumpkin oil, fewer differences with the control sample are observed. Fuets elaborated using only chia and poppy oils show the largest differences with the control sample.

Table 5 shows the results of the consumers’ sensory evaluation of the fuet formulations under study. The findings show that, for most of the attributes, the control samples are rated more highly than the formulations in which pork fat was replaced with seed oils. Nonetheless, the new fuet sausage formulations possess a good overall sensory quality. Specifically, the most highly rated fuets are those prepared with pumpkin seed oils, while those with the lowest scores are those made with poppy seed oil. The use of poppy seed oil in the elaboration of hamburgers has previously generated lower taste scores from consumers [14]. Although pumpkin seed oils have already been analyzed as being a useful functional food for the preparation of meat products [61], our findings suggest they are interesting for food production even when the oils are obtained from agri-food industry waste.

Several authors have reported that adding small amounts of vegetable oils (5%) to fermented sausages increases their sensory ratings [26]. Pires et al. [62] subsequently found reduced sensory ratings for bologna sausages when more than 25% of the animal fat was replaced with vegetable oils. Our findings, broadly speaking, show no significant differences in consumer preferences according to the percentage of substituted animal fat, noting that in all cases the substitutions were of 50% or more. Hence, although substitution does generate lower ratings, this evaluation seems to be largely independent of the percentages of substitution when such levels are very high (+50%).

**Figure 3:**
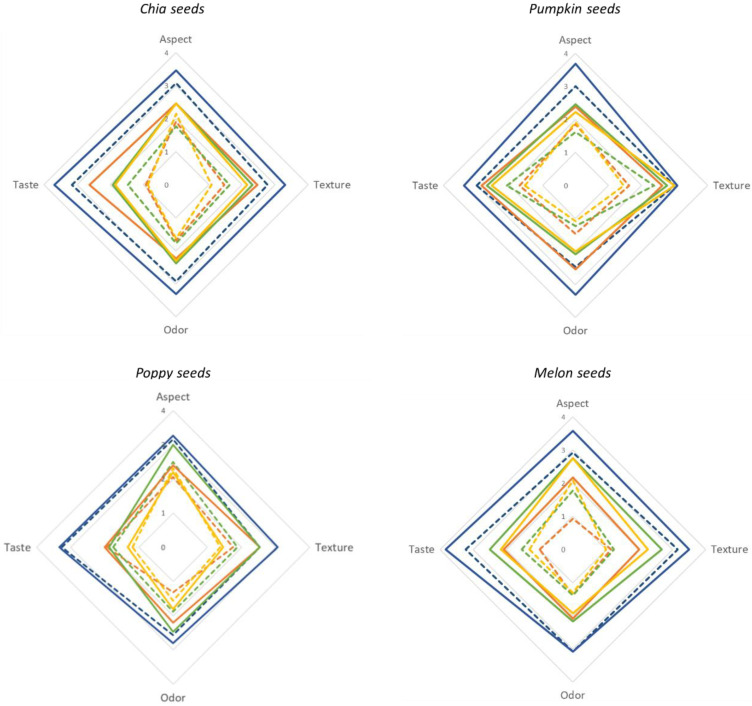
Radar charts for the sensory evaluation of segmented consumers (neophobic and non-neophobic consumers). Solid lines indicate the values of non-neophobic consumers for the proposed attributes and dotted lines values for neophobic consumers. Blue lines indicate the evaluation of the control sample, orange lines the 50% replacement, green lines 75% replacement and yellow lines 100% replacement. The consumers evaluated the physical aspect, texture, odor, and taste using a 9-point scale (–4: extremely dislike; 0: neither like nor dislike; +4: like extremely).

It is also worth underlining the large disparities in our consumers’ ratings, which appear to be a result of their different preferences towards the same fuet sausage. To address this high degree of variability, the consumers were segmented according to their level of neophobia (Figure 3). An analysis of the results shows that the ratings of neophilic (lower neophobia) consumers are systematically higher than those of their neophobic (greater neophobia) counterparts. The greater willingness of neophilic consumers to try, and accept, new foods [32] is presumably the reason for their significantly higher ratings on some of the specific sensory attributes of the novel fuet formulations. Neophobic consumer ratings are smaller for reformulated products, but also for the control sample. The reluctance of neophobic consumers to try new foods seems to result in lower ratings that these consumers provide to any product.

For the case of smoked bacon, Saldaña et al. [63] reported healthiness to be the most important factor for consumers. Hence, the lower ratings in the sensory study might be offset by the higher nutritional quality of the products developed.

Table 6 shows the frequency with which the participants consume fuet and the differences in the frequency of consumption between neophobic and non-neophobic consumers. Regarding the frequency of fuet sausage consumption, our results show a slightly lower rate of consumption compared with previous studies [64]. The segmentation according to the FNSs revealed that consumers with higher levels of food neophobia consume fuet less frequently. Among consumers identified as non-neophobic, 72.5% consume fuet at least once every two weeks, while this percentage is considerably lower in the case of neophobes (30.9%) Previous studies on specific foods have previously established that consumption of a particular product is associated with a lower level of neophobia towards the corresponding food [34]. In our case, as we measured overall food neophobia and not specifically towards fuet, we are unable to corroborate that claim. In Table 5, however, it can be seen that neophobic consumers, who present a significantly lower level of consumption of fuet (Table 6), systematically award lower ratings to many of the attributes on the fuet formulations under analysis.

## 4. Conclusions

The present study demonstrates the possibility of replacing animal fat with vegetable oils to obtain fuet sausages with a lower fat content and a greater presence of unsaturated and polyunsaturated fatty acids, thus leading to a healthier food product. The results show that using oils derived from different seeds generates fuet sausages with higher linoleic acid content and even higher linolenic acid content, which replace the palmitic and stearic acids present in traditional fuet. Thus, these reformulated fuet sausages can be considered healthier than traditional fuet sausages. However, the use of these oils in the percentages analyzed in our study does not result in fuets with a lower calorie content.

Regarding our consumers’ evaluation, it is worth underlining that the results of the sensory analysis show that the substitutions undertaken gave rise to lower ratings. However, the products continued to be positively evaluated. Additionally, as their ratings were lower regardless of the percentage of substitution, it would be advisable to opt for higher percentages of replacement, at least 75%, with the aim of the lower rating being offset by the higher nutritional value of the product. In this sense, it would be necessary to communicate the advantages of these products and create specific identifying markers, such as labels, which would be displayed in the place of purchase to inform consumers in their buying decision processes. Furthermore, these novel, reformulated products should be aimed at the consumer segment with the lowest levels of food neophobia.

Future research should address the growing discussion on consumer acceptance of functional products, that is, products that are healthier, but which, in order to be so, lose some of their organoleptic quality.

## Figures and Tables

**Table 1:** Results obtained for the texture parameters of the fuet samples prepared with different emulsified seed oils.

Sample	Hardness (N)	Stickiness (N⋅mm)	Cohesiveness	Springiness (mm)	Chewiness (N⋅mm)
Control	440.03 ^a^ ± 26.16	−0.48 ^cd^ ± 0.10	0.76 ^a^ ± 0.03	1.00 ^c^ ± 0.18	335.74 ^a^ ± 61.45
Poppy 50%	236.72 ^b^ ± 28.04	−0.92 ^abc^ ± 0.08	0.54 ^cde^ ± 0.02	2.02 ^ab^ ± 0.55	255.37 ^b^± 56.81
Poppy 75%	200.08 ^bc^ ± 9.78	−0.36 ^d^ ±0.05	0.49 ^ef^ ± 0.03	1.90 ^ab^ ± 0.15	186.29 ^b^ ± 10.60
Poppy 100%	199.79 ^cd^ ± 22.65	−0.38 ^d^ ± 0.07	0.48 ^ef^ ± 0.02	1.75 ^abc^ ± 0.14	168.49 ^b^ ± 25.96
Pumpkin 50%	241.81 ^b^ ± 17.17	−1.28 ^a^ ± 0.38	0.50 ^def^ ± 0.03	1.99 ^ab^ ± 0.47	238.66 ^ab^ ± 57.33
Pumpkin 75%	241.81 ^b^ ± 19.93	−0.84 ^abc^ ± 0.20	0.65 ^b^ ± 0.01	1.62 ^bc^ ± 0.53	246.86 ^ab^ ± 80.34
Pumpkin 100%	232.36 ^b^ ± 18.46	−0.66 ^bcd^± 0.14	0.49 ^ef^ ± 0.02	1.79 ^abc^ ± 0.32	204.22 ^b^ ± 37.07
Chia 50%	215.0 ^bc^ ± 16.91	−0.65 ^bcd^ ± 0.28	0.50 ^def^ ± 0.06	1.84 ^ab^ ± 0.57	201.17 ^b^ ± 65.94
Chia 75%	167.26 ^d^ ± 25.34	−0.56 ^cd^ ± 0.24	0.58 ^c^ ± 0.04	2.23 ^ab^ ± 0.37	211.96 ^b^ ± 23.22
Chia 100%	192.15 ^cd^ ± 22.12	−0.52 ^cd^ ± 0.10	0.56 ^cd^ ± 0.03	1.52 ^bc^ ± 0.22	163.45 ^b^ ± 22.07
Melon 50%	208.59 ^bc^ ± 15.41	−1.07 ^ab^ ± 0.33	0.47 ^f^ ± 0.03	2.54 ^a^ ± 0.26	247.16 ^ab^ ± 29.52
Melon 75%	219.17 ^bc^ ± 12.30	−0.68 ^bcd^ ± 0.13	0.46 ^f^ ± 0.03	2.15 ^ab^ ± 0.29	214.25 ^b^ ± 23.17
Melon 100%	183.08 ^cd^ ± 16.71	−0.52 ^cd^ ± 0.27	0.44 ^f^ ± 0.02	1.99 ^ab^ ± 0.22	159.48 ^b^ ± 21.70

Numbers are means with standard errors. Different letters in the same column indicate statistically significant differences (*p* < 0.05).

**Table 2:** Results obtained for the color parameters of the fuet samples prepared with different emulsified seed oils.

Sample	L*	a*	b*	C*	h*
Control	36.53 ^ab^ ± 1.62	12.71 ^d^ ± 1.39	5.08 ^d^ ± 0.89	13.70 ^d^ ± 1.62	21.43 ^b^ ± 1.30
Poppy 50%	39.08 ^a^ ± 1.28	16.07 ^c^ ± 0.33	7.99 ^bc^ ± 0.28	18.06 ^c^ ± 0.50	26.28 ^a^ ± 0.57
Poppy 75%	35.57 ^abc^ ± 2.48	16.03 ^c^ ± 1.11	8.01 ^bc^ ± 0.81	17.92 ^c^ ± 1.29	26.43 ^a^ ± 1.54
Poppy 100%	31.95 ^c^ ± 1.77	15.95 ^c^ ± 0.85	7.80 ^c^ ± 0.68	17.82 ^c^ ± 1.08	25.91 ^a^ ± 1.12
Pumpkin 50%	36.44 ^ab^ ± 1.61	15.54 ^c^ ± 0.57	8.21 ^bc^ ± 0.37	17.58 ^c^ ± 0.49	27.92 ^a^ ± 1.75
Pumpkin 75%	33.99 ^bc^ ± 1.41	15.63 ^c^ ± 1.12	8.24 ^bc^ ± 0.83	17.68 ^c^ ± 1.25	27.77 ^a^ ± 2.07
Pumpkin 100%	35.20 ^abc^ ± 2.21	15.39 ^c^ ± 0.75	8.13 ^bc^ ± 0.28	17.42 ^c^ ± 0.68	27.89 ^a^ ± 1.51
Chia 50%	35.18 ^abc^ ± 1.72	17.21 ^ab^ ± 1.35	8.23 ^bc^ ± 0.77	19.07 ^abc^ ± 1.51	25.45 ^a^ ± 1.15
Chia 75%	35.25 ^abc^ ± 1.73	18.15 ^ab^ ± 0.66	9.31 ^ab^ ± 0.75	20.40 ^ab^ ± 0.92	27.05 ^a^ ± 1.16
Chia 100%	37.49 ^ab^ ± 3.01	18.75 ^a^ ± 0.98	9.92 ^a^ ± 0.73	21.21 ^a^ ± 1.16	27.77 ^a^ ± 1.09
Melon 50%	35.38 ^abc^ ± 1.76	16.52 ^bc^ ± 0.47	8.21 ^bc^ ± 0.53	18.45 ^bc^ ± 0.61	26.32 ^a^ ± 1.16
Melon 75%	36.45 ^ab^ ± 1.30	16.51 ^bc^ ± 0.38	8.14 ^bc^ ± 0.41	18.41 ^bc^ ± 0.45	26.15 ^a^ ± 1.06
Melon 100%	34.45 ^bc^ ± 0.83	16.28 ^bc^ ± 0.93	8.10 ^bc^ ± 0.90	18.62 ^bc^ ± 1.17	27.52 ^a^ ± 1.31

Numbers are means with standard errors. Different letters in the same column indicate statistically significant differences (*p* < 0.05).

**Table 3:** Results of the nutritional evaluation of the fuet samples prepared with different emulsified seed oils.

Sample	H (%)	N (%)	P (%)	Ash (%)	F (%)	CHt (%)	E (Kcal)
Control	8.3 ± 0.35	8.19 ± 0.89	51.19 ± 1.55	8.49 ± 0.67	37.78 ^a^ ± 1.67	2.54 ^d^ ± 0.54	555 ± 78.56
Poppy 50%	7.1 ± 0.76	7.22 ± 0.27	45.13 ± 1.77	7.96 ± 0.27	35.93 ^a^ ± 1.34	10.99 ^b^ ± 0.73	548 ± 65.35
Poppy 75%	6.7 ± 0.25	8.01 ± 0.82	50.06 ± 1.67	8.75 ± 0.57	29.93 ^b^ ± 1.61	11.26 ^b^ ± 0.89	515 ± 61.78
Poppy 100%	8.0 ± 0.77	8.32 ± 0.35	52.00 ± 1.23	10.25 ± 0.45	20.58 ^c^ ± 2.05	16.90 ^a^ ± 0.47	461 ± 34.34
Pumpkin 50%	8.0 ± 0.94	7.06 ± 0.68	44.12 ± 1.78	8.00 ± 0.91	35.02 ^a^ ± 1.81	11.87 ^b^ ± 0.71	539 ± 45.76
Pumpkin 75%	6.0 ± 0.35	7.11 ± 0.35	44.44 ± 1.89	9.16 ± 0.65	34.02 ^ab^ ± 1.32	12.38 ^b^ ± 0.68	533 ± 35.81
Pumpkin 100%	8.4 ± 0.34	7.55 ± 0.94	47.19 ± 1.34	10.29 ± 0.89	29.61 ^b^ ± 2.09	12.91 ^b^ ± 0.91	507 ± 67.38
Chia 50%	8.6 ± 0.36	7.23 ± 0.41	45.19 ± 1.56	8.91 ± 0.87	36.26 ^a^ ± 1.54	9.64 ^c^ ± 0.38	546 ± 52.65
Chia 75%	7.1 ± 0.28	7.59 ± 0.53	47.44 ± 1.36	9.26 ± 0.56	31.30 ^b^ ± 2.81	12.00 ^b^ ± 0.36	525 ± 46.90
Chia 100%	6.4 ± 0.77	7.80 ± 0.33	48.75 ± 1.47	9.54 ± 0.35	24.93 ^c^ ± 1.27	16.78 ^a^ ± 0.81	486 ± 64.34
Melon 50%	7.8 ± 0.75	7.25 ± 0.38	45.31 ± 2.01	8.36 ± 0.45	34.54 ^ab^ ± 1.61	7.79 ^c^ ± 0.54	523 ± 65.54
Melon 75%	7.9 ± 0.73	7.93 ± 0.75	49.56 ± 2.35	8.84 ± 0.45	33.17 ^b^ ± 2.21	8.43 ^c^ ± 0.35	530 ± 73.37
Melon 100%	7.7 ± 0.91	7.98 ± 0.67	49.88 ± 0.99	9.68 ± 1.03	28.69 ^b^ ± 1.83	11.76 ^b^ ± 1.01	505 ± 72.60

Numbers are means with standard errors. (H): % humidity; (N): % total nitrogen; (P): % total protein; (Ash): % ash; (F): % crude fat; (CHt): % total carbohydrates; (E): energy value in kcal/100 g. Different letters in the same column indicate statistically significant differences (*p* < 0.05).

**Table 4:** Results for the composition of the main fatty acids of the fuet samples prepared using different emulsified seed oils.

Sample	Palmitic Acid (%)	Stearic Acid (%)	Oleic Acid (%)	Linoleic Acid (%)	Linolenic Acid (%)
Control	23.30 ^a^ ± 0.22	11.80 ^a^ ± 0.35	45.70 ^a^ ± 1.19	12.60 ^c^ ± 0.36	0.66 ^d^ ± 0.10
Poppy 50%	21.90 ^ab^ ± 0.36	11.30 ^a^ ± 0.27	40.80 ^ab^ ± 1.45	20.20 ^bc^ ± 0.29	0.57 ^d^± 0.05
Poppy 75%	20.00 ^b^ ±0.30	10.10 ^b^ ± 0.32	36.80 ^bc^ ± 0.98	28.30 ^b^ ± 0.33	0.57 ^d^ ± 0.08
Poppy 100%	16.90 ^c^ ± 0.15	7.82 ^c^ ± 0.29	31.00 ^bc^ ± 1.14	40.50 ^a^ ± 1.12	0.63 ^d^ ± 0.07
Pumpkin 50%	22.50 ^ab^ ± 0.51	11.50 ^a^ ± 0.31	42.50 ^ab^ ± 1.33	17.40 ^bc^ ± 0.26	1.09 ^d^ ± 0.28
Pumpkin 75%	19.90 ^b^ ± 0.20	10.90 ^b^ ± 0.18	41.50 ^ab^ ± 1.64	23.00 ^b^ ± 0.74	0.47 ^d^ ± 0.05
Pumpkin 100%	19.60 ^b^ ± 0.13	10.20 ^b^ ± 0.22	35.60 ^bc^ ± 1.32	31.20 ^ab^ ±1.01	0.51 ^d^ ± 0.07
Chia 50%	21.00 ^ab^ ± 0.29	10.70 ^b^ ± 0.21	39.00 ^b^ ± 1.29	12.50 ^c^ ± 0.28	11.90 ^c^ ± 0.37
Chia 75%	19.60 ^b^ ± 0.32	10.50 ^b^ ± 0.25	34.20 ^bc^ ± 1.32	13.80 ^c^ ± 0.34	17.80 ^b^ ± 0.25
Chia 100%	16.30 ^c^ ± 0.25	8.45 ^c^ ± 0.19	27.30 ^c^ ± 1.92	16.20 ^bc^ ± 0.26	28.40 ^a^ ± 0.36
Melon 50%	22.30 ^ab^ ± 0.34	11.90 ^a^ ± 0.33	44.70 ^a^ ± 1.06	15.70 ^bc^ ± 0.42	0.40 ^d^ ± 0.03
Melon 75%	19.90 ^b^ ± 0.19	10.90 ^b^ ± 0.26	41.50 ^ab^ ± 0.97	23.00 ^b^ ± 0.56	0.47 ^d^ ± 0.12
Melon 100%	17.00 ^c^ ± 0.23	9.42 ^bc^ ± 0.18	39.50 ^b^ ± 0.85	30.40 ^ab^ ± 1.21	0.45 ^d^ ± 0.16

Numbers are means with standard errors. Different letters in the same column indicate statistically significant differences (*p* < 0.05).

**Table 5:** Sensory evaluation of the fuet sausage samples.

Sample	Aspect	Texture	Odor	Taste
Control	3.22 + 1.12 ^a^	2.81 + 1.14 ^a^	2.68 + 1.30 ^a^	3.29 + 1.01 ^a^
Poppy 50%	2.13 + 1.28 ^b^	2.13 + 1.36 ^b^	1.74 + 1.39 ^b^	1.97 + 1.14 ^b^
Poppy 75%	2.64 + 1.13 ^ab^	2.19 + 1.40 ^ab^	2.16 + 1.32 ^ab^	1.81 + 1.38 ^bc^
Poppy 100%	2.16 + 1.26 ^b^	1.42 + 1.15 ^c^	1.68 + 1.45 ^b^	1.26 + 1.34 ^c^
Control	3.35 + 0.80 ^a^	3.04 + 1.00 ^a^	2.88 + 0.91 ^a^	3.19 + 0.85 ^a^
Pumpkin 50%	2.12 + 1.28 ^b^	2.12 + 1.27 ^b^	2.00 + 1.27 ^b^	2.27 + 1.22 ^b^
Pumpkin 50%	2.12 + 1.28 ^b^	2.12 + 1.27 ^b^	2.00 + 1.27 ^b^	2.27 + 1.22 ^b^
Pumpkin 75%	2.04 + 1.28 ^b^	2.58 + 0.95 ^ab^	1.65 + 1.23 ^b^	2.38 + 1.20 ^b^
Pumpkin 100%	2.08 + 1.32 ^b^	2.23 + 1.39 ^b^	1.54 + 1.27 ^b^	2.04 + 1.37 ^b^
Control	3.27 + 0.83 ^a^	3.04 + 1.00 ^a^	3.12 + 0.99 ^a^	3.42 + 0.81 ^a^
Chia 50%	2.19 + 1.27 ^b^	1.96 + 1.22 ^b^	1.96 + 1.11 ^b^	1.77 + 1.24 ^b^
Chia 75%	2.12 + 1.14 ^b^	1.96 + 1.11 ^b^	2.08 + 1.32 ^b^	1.69 + 1.09 ^b^
Chia 100%	2.31 + 1.35 ^b^	1.62 + 1.30 ^b^	1.96 + 1.22 ^b^	1.35 + 1.20 ^b^
Control	3.24 + 1.01 ^a^	3.32 + 0.90 ^a^	3.08 + 1.08 ^a^	3.52 + 0.96 ^a^
Melon 50%	1.52 + 1.48 ^c^	1.56 + 1.23 ^b^	1.71 + 1.37 ^b^	1.52 + 1.39 ^b^
Melon 75%	2.24 + 1.01 ^b^	1.92 + 1.38 ^b^	1.76 + 1.20 ^b^	2.00 + 1.19 ^b^
Melon 100%	2.40 + 1.19 ^b^	1.60 + 1.35 ^b^	1.60 + 1.26 ^b^	1.72 + 1.24 ^b^

Numbers are means with standard errors. Different letters in the same column for each attribute indicate statistically significant differences (*p* < 0.05).

**Table 6:** Consumer segmentation using the Food Neophobia Scale (FNS).

Frequency	Total Consumers Frequency	Consumer Segmentation	
		Neophobic	Non-Neophobic
Once a week	28.0%	21.4%	35.0%
Once a fortnight	23.2%	9.5%	37.5%
Once a month	22.0%	21.4%	22.5%
Once every two month or less	26.8%	47.6%	5.0%

Chi squared values for consumption frequency are χ^2^ = 22,147, *df* = 3, *p* = 0.000.

## Data Availability

Data available on request.
